# Optical coherence tomography findings in Duchenne muscular dystrophy

**DOI:** 10.1186/s12886-026-04756-2

**Published:** 2026-03-24

**Authors:** Gökhan Yöyler, Seda Karaca Adıyeke

**Affiliations:** 1 Aliaga State Hospital, Izmir, Türkiye; 2 Department of Ophthalmology, Izmir Bakırçay University School of Medicine, Izmir, Türkiye

**Keywords:** Duchenne muscular dystrophy, Optical coherence tomography, Ganglion cell–inner plexiform layer, Inner nuclear layer, Vitreoretinal interface

## Abstract

**Purpose:**

To evaluate vitreoretinal interface (VRI) findings and layer-specific macular thickness profiles on optical coherence tomography (OCT) in children with Duchenne muscular dystrophy (DMD).

**Methods:**

This retrospective case–control study included 26 children with DMD and 28 age- and sex-matched healthy controls. All participants underwent comprehensive ophthalmic examination and Spectralis spectral-domain OCT. Ganglion cell–inner plexiform layer (GCIPL), inner nuclear layer (INL), and outer plexiform layer (OPL) thicknesses were obtained using the ETDRS grid (central 1-mm subfield and quadrants of the 3-mm and 6-mm rings). Between-group comparisons were evaluated using covariate-adjusted models (age, spherical equivalent, and intraocular pressure), with false discovery rate (Benjamini–Hochberg) correction applied within each layer. Only right eyes were analyzed.

**Results:**

Best-corrected visual acuity was 0.0 logMAR in both groups, and spherical equivalent and intraocular pressure were comparable. No VRI abnormalities or vascular manifestations, including Duchenne-associated proliferative retinopathy, were detected. After covariate adjustment and FDR correction, GCIPL thickness remained reduced in the 3-mm inferior and 6-mm temporal sectors (both q = 0.0135), and INL thickness remained reduced in the 3-mm inferior sector (q = 0.036). Within the DMD group, age was not significantly correlated with these parameters.

**Conclusion:**

Children with DMD and preserved visual acuity showed subtle, sector-specific thinning of inner retinal layers (GCIPL and INL) on OCT, while VRI and vascular abnormalities were absent. Larger longitudinal studies are needed to confirm these findings and clarify their clinical significance.

## Introduction

Duchenne muscular dystrophy (DMD) is the most common X-linked recessive neuromuscular disorder and typically presents in early childhood with progressive proximal muscle weakness [1, 2]. The disease is caused by pathogenic variants in the dystrophin gene, most frequently large deletions [1, 2]. Beyond its well-established effects on skeletal, cardiac, and respiratory muscles, dystrophin deficiency also has important implications for the nervous system. Dystrophin isoforms contribute to neural development and synaptic function, including the maturation of neurotransmitter–receptor complexes and the regulation of excitatory–inhibitory signaling [3–7]. Accordingly, dystrophin-related pathology may extend beyond muscle tissue to involve both the peripheral and central nervous systems, including the retina [8, 9].

Within the retina, dystrophin and its isoforms have been localized to photoreceptor terminals, synaptic layers, Müller cells, the ganglion cell layer, and the inner limiting membrane [8, 10–14]. While many reports have emphasized functional involvement—particularly electrophysiological alterations—structural characterization of retinal involvement in DMD remains comparatively limited. Emerging clinical evidence, including a recent review by Barboni et al., suggests that a subset of patients may develop severe ocular manifestations such as Duchenne-associated proliferative retinopathy (DAPR) [15]. These observations underscore the potential value of detailed retinal imaging to detect subclinical structural changes and to further refine our understanding of retinal involvement in DMD.

Optical coherence tomography (OCT) enables noninvasive, high-resolution assessment of the vitreoretinal interface and quantitative analysis of individual retinal layers. In this study, we aimed to evaluate vitreoretinal interface findings and layer-specific thickness measurements—focusing on the outer plexiform layer (OPL), inner nuclear layer (INL), and ganglion cell–inner plexiform layer (GCIPL) complex—to further characterize structural features that may accompany dystrophin deficiency.

## Methods

### Study design and participants

This retrospective case–control study was based on a review of the medical records of patients with Duchenne muscular dystrophy (DMD) who were referred from the pediatric neurology clinic between January 2018 and February 2021. The study adhered to the principles of the Declaration of Helsinki and was approved by the Ethics Committee of the University of Health Sciences, Hamidiye Faculty of Medicine (Approval No: 226; Date: 20.11.2020). Although the study was retrospective and based on previously recorded clinical data, written informed consent was obtained from the parents or legal guardians of all participants, since all participants were younger than 16 years.

An age- and sex-matched healthy control group was established from children presenting to the pediatric ophthalmology department for routine vision screening or refractive evaluation. All participants underwent a comprehensive ophthalmological examination, including best-corrected visual acuity (BCVA) assessment using Snellen charts, slit-lamp biomicroscopy, intraocular pressure (IOP) measurement, and dilated fundus examination.

Exclusion criteria for all participants included spherical refractive error > 5.0 diopters (D) or astigmatism > 3.0 D, a history of ocular surgery or trauma, and pre-existing ocular pathology (including retinal or optic nerve abnormalities). Healthy controls were excluded if they had any systemic disease or a history of medication use—particularly systemic corticosteroids—known to influence retinal morphology. Participants with cognitive impairment precluding reliable visual acuity assessment were excluded. Only individuals with normal ophthalmic findings and OCT scans meeting the predefined image quality threshold (signal strength ≥ 20 dB) were included.

Comprehensive genetic profiles were available for all patients with DMD. However, given the heterogeneity of mutations and the modest sample size, the study was not sufficiently powered to evaluate genotype–phenotype associations or isoform-specific retinal patterns. Therefore, quantitative OCT metrics were analyzed at the cohort level rather than stratified by genotype or predicted dystrophin isoform involvement.

### OCT imaging

All OCT examinations were performed by the same experienced technician using a spectral-domain OCT device (Spectralis OCT; Heidelberg Engineering, Heidelberg, Germany) following the standardized scanning protocol of our clinic. Macular imaging consisted of 49 B-scans (512 A-scans) acquired at 120-µm intervals within a 20° × 20° field, with each B-scan averaged from nine images. The fovea was additionally assessed using single horizontal and vertical high-resolution line scans, each averaged from 100 images.

### Retinal layer segmentation and thickness measurements

Automated retinal layer segmentation was performed using the device’s built-in software. All B-scans were subsequently reviewed for segmentation errors by a single expert retina specialist (S.K.A.), who was masked to group assignment, and manual corrections were performed when necessary. Only OCT scans meeting the predefined image quality threshold (signal strength ≥ 20 dB) were included in both groups. Mean thickness values for the GCIPL, INL, and OPL were extracted using the Early Treatment Diabetic Retinopathy Study (ETDRS) grid centered on the fovea. Measurements were recorded for the central 1-mm subfield and for the superior, inferior, nasal, and temporal quadrants of the inner (3-mm) and outer (6-mm) rings.

### Statistical analysis

All statistical analyses were performed using IBM SPSS Statistics, version 23.0 (IBM Corp., Armonk, NY, USA). Continuous variables are presented as mean ± standard deviation (SD) or median (interquartile range [IQR]) based on distribution, and categorical variables as counts and percentages. Distributional assumptions were evaluated using the Kolmogorov–Smirnov test and visual inspection of histograms.

To avoid inter-eye correlation and ensure independent observations, only the right eye of each participant was included in the analyses. Baseline demographic and clinical variables were compared between groups using the independent-samples t test for normally distributed variables and the Mann–Whitney U test for non-normally distributed variables.

For OCT layer thickness outcomes, unadjusted between-group comparisons were first performed using the same approach. To address potential confounding by refractive status and IOP—as well as age-related variation in pediatric retinal thickness—adjusted between-group analyses were conducted using analysis of covariance (ANCOVA) models, with sectoral layer thickness as the dependent variable, group (DMD vs. control) as the main factor, and age, spherical equivalent, and intraocular pressure as covariates. Adjusted group effects are reported as the regression coefficient for group (β) with 95% confidence intervals (CI), along with the corresponding covariate-adjusted p values (p_adj).

Because multiple sectoral comparisons were performed within each retinal layer (central 1-mm subfield and the four quadrants of the 3-mm and 6-mm rings), multiplicity was controlled using the Benjamini–Hochberg false discovery rate (FDR) procedure applied within each layer (GCIPL, INL, and OPL). FDR-corrected q values are reported, and statistical significance after multiplicity correction was defined as q < 0.05; findings not surviving FDR correction were interpreted as exploratory.

Within the DMD group, correlations between age and retinal thickness parameters that remained significant after covariate-adjusted, FDR-corrected between-group comparisons were assessed using Pearson correlation coefficients (r) with two-sided p values. All statistical tests were two-sided.

## Results

Initially, 43 patients with DMD and 41 healthy controls were reviewed. After applying the predefined image quality threshold (signal strength ≥ 20 dB), OCT scans from 31 patients in the DMD group (72.1%) and 28 participants in the control group (68.3%) were eligible for quantitative analysis. The proportion of excluded scans due to insufficient signal strength was similar between groups. Among the 31 eligible DMD patients, three exhibited peripheral pigmentary changes and two had optic disc drusen on fundus examination. Because these ocular abnormalities could potentially influence retinal morphology and OCT-derived measurements, these five patients were excluded a priori. Consequently, the final analysis included 26 patients with DMD (Group 1) and 28 age- and sex-matched healthy volunteers (Group 2) without fundus pathology.

The mean age was 9.36 ± 2.50 years (range, 5–14) in Group 1 and 9.96 ± 2.18 years (range, 7–14) in Group 2. All participants in both groups demonstrated a best-corrected visual acuity of 0.0 logMAR. The mean spherical equivalent refractive error was 0.19 ± 0.57 diopters (range, + 0.75 to − 1.25) in Group 1 and 0.15 ± 0.46 diopters (range, + 0.50 to − 0.75) in Group 2, with no statistically significant difference between groups (*p* = 0.82). Similarly, mean intraocular pressure was 14.3 ± 2.8 mmHg (range, 10–17) in Group 1 and 13.8 ± 3.4 mmHg (range, 9–18) in Group 2 (*p* = 0.76).

Regarding the genetic mutation spectrum of the DMD cohort, the most prevalent mutations were exon deletions (*n* = 19; 73.1%), followed by duplications (*n* = 5; 19.2%) and nonsense mutations (*n* = 2; 7.7%). Although comprehensive genetic data were available, heterogeneity of mutation types and the limited sample size precluded formal genotype–phenotype correlation or isoform-specific analyses; therefore, all quantitative retinal parameters were reported at the aggregate cohort level. The mutation spectrum is summarized in Table 1.

**Table 1:** Distribution of dystrophin gene mutations and predicted affected isoforms in the Duchenne muscular dystrophy (DMD) cohort (*n* = 26)

Mutation Type	Exon Location	Patients (*n*)	Predicted Affected Isoforms
Deletions (*n* = 19)	3–7, 5–7, 8–13	4	Dp427
	43–45, 45, 47–48, 48–50, 50, 51–54	10	Dp427, Dp260
	48–52, 45–52	4	Dp427, Dp260, Dp140
	70	1	Dp427, Dp260, Dp140, Dp71
Duplications (*n* = 5)	2, 3–7	4	Dp427
	56–57	1	Dp427, Dp260, Dp140
Nonsense (*n* = 2)	70	2	Dp427, Dp260, Dp140, Dp71
**Total**		**26**	

In the OPL, sectoral thickness measurements are summarized in Table 2. In unadjusted analyses, a small between-group difference was observed only in the 3-mm temporal quadrant, where OPL thickness was greater in the DMD group than in controls (36.8 ± 2.4 μm [range, 32–42] vs. 34.3 ± 2.8 μm [range, 28–40]; *p* = 0.046). However, after covariate adjustment using ANCOVA (age, spherical equivalent, and intraocular pressure), this difference was no longer statistically significant (p_adj = 0.12) and did not survive false discovery rate correction within the OPL layer (q = 0.18). No other OPL sector in the central 1-mm subfield or within the 3-mm and 6-mm rings showed a significant between-group difference in the adjusted analyses (all p_adj ≥ 0.24) or after FDR correction (all q ≥ 0.36). Accordingly, OPL findings were considered exploratory.

**Table 2:** Comparison of outer plexiform layer (OPL) thickness (µm) between Duchenne muscular dystrophy patients (Group 1) and healthy controls (Group 2) measured by spectral-domain OCT

	Group 1 (*n* = 26)	Group 2 (*n* = 28)	Unadjusted *P* value	p_adj	q (FDR)
**OPL**					
** 1 mm**	26.1 ± 2.4 (20–29)	26.4 ± 2.1 (22–31)	0.670	0.75	0.78
** 3 mm**					
Temporal	36.8 ± 2.4 (32–42)	34.3 ± 2.8 (28–40)	0.046	0.12	0.18
Inferior	37.1 ± 2.8 (31–44)	38.4 ± 3.2 (32–45)	0.670	0.762	0.84
Nasal	33.1 ± 3.9 (25–42)	32.7 ± 3.5 (24–42)	0.960	0.98	0.98
Superior	35.9 ± 2.8 (29–41)	34.2 ± 2.7 (29–39)	0.560	0.68	0.72
** 6 mm**					
Temporal	29.7 ± 2.6( 25–34)	28.4 ± 3.1 (23–34)	0.160	0.24	0.36
Inferior	33.1 ± 2.8 (28–38)	33.5 ± 3 (31–40)	0.840	0.92	0.96
Nasal	38.4 ± 3.2 (32–44)	38.1 ± 2.3 (34–42)	0.720	0.76	0.84
Superior	27.8 ± 1.8 (25–31)	28.7 ± 2.1 (25–32)	0.600	0.72	0.78

Values are presented as mean ± SD (range). Unadjusted p values were obtained using independent samples t-test or Mann–Whitney U test, as appropriate. p_adj indicates p values from ANCOVA adjusted for age, spherical equivalent, and intraocular pressure. q values indicate false discovery rate (Benjamini–Hochberg)–corrected results within the OPL layer. Statistical significance after multiplicity correction was defined as q < 0.05

In the GCIPL, sectoral thickness values are summarized in Table 3. In unadjusted analyses, GCIPL thickness appeared lower in the DMD group than in controls in the 3-mm temporal (*p* = 0.042), inferior (*p* = 0.012), and superior sectors (*p* = 0.014), as well as in the 6-mm temporal sector (*p* = 0.002). After covariate adjustment using ANCOVA (age, spherical equivalent, and intraocular pressure) and false discovery rate correction within the GCIPL layer, statistically significant between-group differences persisted only in the 3-mm inferior sector (47.4 ± 4.7 vs. 51.5 ± 6.1 μm; p_adj = 0.0015; q = 0.0135; β=−2.8 μm, 95% CI − 3.2 to − 0.8) and the 6-mm temporal sector (31.7 ± 2.6 vs. 34.8 ± 3.8 μm; p_adj = 0.003; q = 0.0135; β=−2.3 μm, 95% CI − 2.4 to − 0.7). The remaining GCIPL sectors did not demonstrate FDR-corrected significance (all q ≥ 0.063) and were therefore considered exploratory.

**Table 3:** Comparison of ganglion cell–inner plexiform layer (GCIPL) thickness (µm) measured by spectral-domain OCT between patients with Duchenne muscular dystrophy (Group 1) and healthy controls (Group 2)

	Group 1 (*n* = 26)	Group 2 (*n* = 28)	Unadjusted *P* value	p_adj	q (FDR)
**GCIPL**					
** 1 mm**	15.7 ± 2.8 (11–19)	16.1 ± 3.9 (11–23)	0.850	0.92	0.960
** 3 mm**					
Temporal	45.7 ± 4.5(38–52)	48.4 ± 5.3 (43–56)	0.042	0.560	0.960
Inferior	47.4 ± 4.7 (40–55)	51.5 ± 6.1 (45–59)	0.012	0.0015	0.0135*
Nasal	49.8 ± 6.3( 43–56)	50.7 ± 4.5 (44–57)	0.138	0.240	0.432
Superior	47.9 ± 5.6 (41–58)	52.3 ± 6.2 (44–61)	0.014	0.021	0.0630
** 6 mm**					
Temporal	31.7 ± 2.6 (29–44)	34.8 ± 3.8 (31–48)	0.002	0.003	0.0135*
Inferior	31.4 ± 3.5 (28–35)	32.6 ± 3.4 (27–39)	0.124	0.196	0.432
Nasal	34.6 ± 3.7 (27–41)	35.4 ± 4.8 (30–42)	0.56	0.84	0.840
Superior	37.9 ± 5.1 (32–41)	38.4 ± 4.1(32–41)	0.836	0.960	0.920

* Values are presented as mean ± SD (range). Unadjusted p values were calculated using the independent-samples t test or the Mann–Whitney U test, as appropriate. p_adj denotes p values from ANCOVA adjusted for age, spherical equivalent, and intraocular pressure. q values represent Benjamini–Hochberg false discovery rate (FDR)–corrected results within the GCIPL layer. Statistical significance after multiplicity correction was defined as q < 0.05

In the INL, sectoral thickness values are summarized in Table 4. In unadjusted analyses, INL thickness was lower in the DMD group than in controls in the 3-mm temporal sector (36.2 ± 3.7 vs. 39.1 ± 4.3 μm; *p* = 0.041), the 3-mm inferior sector (24.3 ± 3.3 vs. 28.0 ± 2.1 μm; *p* = 0.003), and the 6-mm inferior sector (31.4 ± 3.9 vs. 34.2 ± 4.4 μm; *p* = 0.014). After covariate adjustment using ANCOVA (age, spherical equivalent, and intraocular pressure) and false discovery rate correction within the INL layer, statistically significant between-group differences persisted only in the 3-mm inferior sector (24.3 ± 3.3 vs. 28.0 ± 2.1 μm; p_adj = 0.004; q = 0.036; β=−2.9 μm, 95% CI − 3.7 to − 1.2). In contrast, the 3-mm temporal and 6-mm inferior sectors did not retain FDR-corrected significance (p_adj = 0.072, q = 0.216 and p_adj = 0.024, q = 0.108, respectively), and no significant between-group differences were observed in the remaining INL sectors after adjustment and multiplicity correction.

**Table 4:** Comparison of inner nuclear layer (INL) thickness (µm) measured by spectral-domain OCT between patients with Duchenne muscular dystrophy (Group 1) and healthy controls (Group 2)

	Group 1 (*n* = 26)	Group 2 (*n* = 28)	Unadjusted *P* value	p_adj	q (FDR)
**INL**					
** 1 mm**	21.6 ± 3.1 (16–27)	22.4 ± 2.3(17–29)	0.850	0.890	0.950
** 3 mm**					
Temporal	36.2 ± 3.7(28–47)	39.1 ± 4.3(31–50)	0.041	0.072	0.216
Inferior	24.3 ± 3.3 (17–32)	28 ± 2.1 (21–34)	0.003	0.004	0.036*
Nasal	38.1 ± 5.1 (28–48)	39 ± 3.1 (34–47)	0.370	0.540	0.810
Superior	37.8 ± 4.7 ( 28–49)	38 ± 4.1( 29–51)	0.120	0.260	0.585
** 6 mm**					
Temporal	35.7 ± 4.2 (29–44)	36.8 ± 2.9 (30–48)	0.960	0.960	0.960
Inferior	31.4 ± 3.9 (25–41)	34.2 ± 4.4 (27–43)	0.014	0.024	0.108
Nasal	37.8 ± 3.6 (29–46)	38.3 ± 4.1 (28–47)	0.360	0.48	0.810
Superior	27.9 ± 3.1 (20–35)	28.6 ± 2.9 (22–35)	0.760	0.840	0.960

* Values are presented as mean ± SD (range). Unadjusted p values were calculated using the independent-samples t test or the Mann–Whitney U test, as appropriate. p_adj denotes p values from ANCOVA adjusted for age, spherical equivalent, and intraocular pressure. q values represent Benjamini–Hochberg false discovery rate (FDR)–corrected results within the INL layer (computed across all INL sectors using p_adj values). Statistical significance after multiplicity correction was defined as q < 0.05; * indicates q < 0.05

Correlation analyses were performed to explore the association between age and the retinal thickness parameters that remained significant after covariate-adjusted, FDR-corrected between-group comparisons. As shown in Table 5, age was not significantly correlated with GCIPL thickness in the 3-mm inferior sector (*r* = − 0.256, *p* = 0.072), GCIPL thickness in the 6-mm temporal sector (*r* = − 0.312, *p* = 0.057), or INL thickness in the 3-mm inferior sector (*r* = − 0.315, *p* = 0.082) within the DMD group. Overall, these analyses did not demonstrate a statistically significant age–thickness association for the robust parameters within this cohort.

**Table 5:** Correlation between age and retinal thickness parameters that remained significant after covariate-adjusted, FDR-corrected between-group comparisons in the DMD group (*n* = 26)

Retinal Layer / Sector	*r*	*p*-value
GCIPL, 3-mm ring (inferior)	–0.256	0.072
GCIPL, 6-mm ring (temporal)	–0.312	0.057
INL, 3 mm ring (inferior)	–0.315	0.082

Pearson correlation coefficient (r). GCIPL: ganglion cell–inner plexiform layer; INL: inner nuclear layer. Correlation analyses were limited to parameters that remained significant in between-group ANCOVA models (with age, spherical equivalent, and intraocular pressure as covariates) after within-layer FDR correction (q < 0.05)

OCT examination revealed no vitreoretinal interface (VRI) abnormalities, and retinal layer architecture appeared regular in all eyes.

## Discussion

Our study was premised on the notion that dystrophin deficiency may be accompanied by subtle, layer-specific retinal alterations, potentially reflecting disruption of synaptic organization and neuroglial homeostasis. Within this framework, OCT demonstrated a pattern of differences in GCIPL and INL thickness between patients with DMD and healthy controls that remained evident after adjustment for key ocular covariates and control for multiplicity. By contrast, the modest between-group signal observed in the OPL in unadjusted comparisons did not persist after covariate adjustment and false discovery rate (FDR) control and should therefore be interpreted with caution. Taken together, these findings suggest that although retinal thickness measures in DMD have often been reported within ranges considered normal in prior studies [16], OCT may capture subtle, layer-dependent structural differences that warrant confirmation in larger, longitudinal cohorts.

In the OPL, a modest between-group signal was observed in unadjusted sectoral comparisons; however, this difference did not persist after covariate adjustment and FDR control, and OPL-related observations should therefore be interpreted cautiously. The OPL remains biologically relevant in DMD, as dystrophin isoforms Dp427 and Dp260 are localized to this layer and have been implicated in the stabilization of ribbon synapses between photoreceptor terminals and bipolar cell dendrites [12, 17, 18]. Experimental studies have described “invagination failure” during the entry of bipolar cell dendrites into photoreceptor terminals in the setting of dystrophin deficiency [19], and apoptosis-related tissue changes have also been discussed [20]. Given that our OPL finding did not survive covariate adjustment and FDR-based multiplicity control,, these mechanistic considerations are presented as hypothesis-generating and warrant evaluation in larger, longitudinal studies.

In contrast, the sectoral reduction in GCIPL and INL thickness observed in our cohort is consistent with inner retinal involvement in DMD and may reflect structural effects of dystrophin deficiency on neuronal and glial elements within these layers. Several dystrophin isoforms, including Dp427, Dp260, and Dp140, are expressed at both somatic and synaptic sites in the retina and have been implicated in maintaining synaptic architecture and cellular stability [12, 21–23]. Although Dp71 is thought to contribute to retinal homeostasis through Müller cell–mediated mechanisms, particularly via the organization of Kir4.1 and AQP4 channel complexes, only a minority of our cohort carried mutations involving regions typically associated with this isoform [15, 24]. Importantly, our study was not designed or powered to establish isoform-specific structure–function relationships; therefore, these considerations provide biological context rather than mechanistic proof. Experimental data nevertheless support the plausibility of inner retinal vulnerability, as Catalani et al. reported that loss of functional Dp427 may alter the balance between apoptosis and autophagy and increase neuronal cell death in the inner retina [20]. Furthermore, developmental studies by Persiconi and colleagues reported that reduced expression of synaptogenesis-related genes in dystrophin deficiency may be associated with delayed maturation of inner retinal networks, which may be relevant to structural differences detected in the GCIPL and INL on OCT [25].

Experimental work has also described changes in inhibitory interneuron populations, including reduced GABAergic amacrine cell density, and metabolic alterations within the ganglion cell layer in dystrophin-deficient models [25, 26]. In addition, impaired trophic signaling and reduced nerve growth factor sensitivity have been proposed as mechanisms that may limit adaptive responses to cellular stress in dystrophin-deficient neurons [26, 27]. Taken together, these data provide biological context for inner retinal vulnerability in DMD; however, our cross-sectional OCT findings cannot disentangle developmental influences from neuroglial or degenerative mechanisms, and mechanistic inferences should therefore be made cautiously.

The DMD gene encodes multiple tissue- and cell-specific dystrophin isoforms (including Dp427, Dp260, Dp140, and Dp71), and prior work suggests that mutation location may influence the pattern and severity of retinal involvement [28]. In our cohort, a minority of patients (11.5%) had distal mutations involving exon 70; however, the modest sample size and heterogeneity of mutation loci precluded statistically powered genotype–phenotype or isoform-specific stratification [29]. Accordingly, we were unable to delineate the relative contributions of individual isoforms to the OCT findings observed in this study. Larger multicenter studies with genetically stratified cohorts would be valuable to clarify potential genotype–phenotype associations and isoform-specific structural signatures.

Sectoral differences in retinal layer thickness may reflect the non-uniform distribution of retinal cell types and synaptic architecture, as well as layer-specific roles of dystrophin isoforms in neuronal and glial compartments. Dystrophin isoforms have been implicated in synaptic organization, neuronal integrity, and glial function in experimental studies [10, 20, 25]. In our cohort, the most robust between-group differences after covariate adjustment and multiplicity control were confined to selected GCIPL and INL sectors. Although the preferential involvement of temporal and inferior regions may relate to regional retinal heterogeneity, including variation in cellular composition and metabolic demand, the present cross-sectional data do not allow firm inferences regarding region-specific vulnerability, and these patterns require confirmation in larger, longitudinal studies.

Sectoral OCT analyses inherently increase the risk of spurious findings when multiple regions are compared. Accordingly, we interpreted the results conservatively and focused on differences that remained robust after adjustment and correction for multiple comparisons. Under this approach, the observed changes were confined to selected GCIPL and INL sectors rather than being widespread across the macula, whereas several nominal signals did not remain robust after correction. Therefore, the spatial pattern should be regarded as hypothesis-generating rather than definitive evidence of region-specific vulnerability. Nonetheless, these findings support the potential of OCT to detect subtle, regionally confined structural differences in DMD even when visual acuity is preserved.

No vascular abnormalities, Duchenne-associated proliferative retinopathy (DAPR), or vitreoretinal interface (VRI) anomalies were identified in the present cohort, which contrasts with some prior reports [30, 31]. The absence of DAPR may be related to the pediatric age range of our participants, as DAPR has been predominantly described in older patients and in more advanced stages of disease. In addition, previous reports have suggested that neovascular responses in DMD may occur in the context of secondary systemic factors such as chronic hypoxia or hypercapnia associated with late-stage cardiopulmonary compromise [32]. Although detailed longitudinal systemic assessments were not available in this retrospective cohort, the lack of clinically apparent advanced systemic involvement may have reduced the likelihood of such vascular phenotypes. Finally, only a minority of our patients carried distal mutations involving regions typically associated with the Dp71 isoform, which has been implicated in blood–retinal barrier homeostasis, potentially further lowering the probability of vascular manifestations in a small pediatric sample. Visual acuity was preserved across the cohort, consistent with the absence of overt vascular or vitreoretinal interface abnormalities.

While age-related changes in retinal thickness have been reported in healthy children and in various neuromuscular disorders [33, 34], we did not observe statistically significant age–thickness correlations for the OCT parameters that remained robust after covariate adjustment and multiplicity control. Within this pediatric cohort, the observed GCIPL and INL differences were not clearly explained by age. Nevertheless, given the modest sample size and the restricted age range, small age-related effects cannot be excluded.

Since systemic corticosteroids are standard therapy in DMD, their potential impact on retinal morphology should be considered. Prolonged steroid exposure may increase intraocular pressure (IOP) and thereby indirectly influence OCT-derived retinal thickness measurements [35]. In our cohort, mean IOP was within normal limits and comparable between the DMD and control groups, suggesting that overt steroid-related ocular hypertension was unlikely to fully explain the observed findings. However, normal IOP does not exclude IOP-independent retinal effects of systemic steroids reported in the literature [36, 37]. Because detailed information on steroid type, cumulative dose, and duration was not available for robust adjustment in this retrospective study, residual confounding related to steroid exposure cannot be ruled out. In addition, refractive status—particularly spherical equivalent (SE)—is a recognized confounder in OCT morphometry [38, 39]; accordingly, SE was included as a covariate in the adjusted analyses to minimize refractive bias.

This study has several limitations. Its retrospective design limits control over systemic confounders, most notably systemic corticosteroid exposure, and the modest sample size precluded statistically powered stratification by mutation locus or predicted dystrophin isoform involvement. Although comprehensive genetic data were available and a minority of patients carried distal mutations (including exon 70), we were unable to delineate isoform-specific contributions to the OCT findings, and our results should therefore be interpreted at the cohort level. In addition, we applied a stringent OCT image-quality criterion (signal strength ≥ 20 dB) and excluded scans with segmentation errors to ensure measurement reliability; however, these quality-control steps reduced the number of analyzable examinations and may limit generalizability. Larger multicenter studies with genetically stratified cohorts, standardized treatment exposure data, and longitudinal OCT follow-up would be valuable to clarify potential genotype–phenotype associations and longitudinal retinal structural changes in DMD.

In conclusion, our findings indicate that pediatric patients with DMD may exhibit subtle, sector-specific structural differences in inner retinal layers, particularly within the GCIPL and INL, despite preserved visual acuity and the absence of vitreoretinal interface or vascular abnormalities. These observations are consistent with the possibility of dystrophin-related retinal involvement, but mechanistic interpretations require confirmation. Larger prospective studies incorporating standardized treatment exposure data, longitudinal OCT follow-up, and genotype-stratified analyses are warranted to validate these results and clarify their clinical implications.

## Data Availability

The datasets used and/or analysed during the current study are available from the corresponding author on reasonable request.

## References

[1] Miyatake M, Miike T, Zhao JE, Yoshioka K, Uchino M, Usuku G. Dystrophin: localization and presumed function. Muscle Nerve. 1991;14(2):113–9. 10.1002/mus.880140205. 1825695 10.1002/mus.880140205

[2] Yiu EM, Kornberg AJ. Duchenne muscular dystrophy. J Paediatr Child Health. 2015;51(8):759–64. 10.1111/jpc.12868. 25752877 10.1111/jpc.12868

[3] Hendriksen RG, Hoogland G, Schipper S, Hendriksen JG, Vles JS, Aalbers MW. A possible role of dystrophin in neuronal excitability: a review of the current literature. Neurosci Biobehav Rev. 2015;51:255–62. 10.1016/j.neubiorev.2015.01.023. 25677308 10.1016/j.neubiorev.2015.01.023

[4] Rae MG, O’Malley D. Cognitive dysfunction in Duchenne muscular dystrophy: a possible role for neuromodulatory immune molecules. J Neurophysiol. 2016;116(3):1304–15. 10.1152/jn.00248.2016. 27385793 10.1152/jn.00248.2016 PMC5023417

[5] Aittaleb M, Martinez-Pena Y, Valenzuela I, Akaaboune M. Spatial distribution and molecular dynamics of dystrophin glycoprotein components at the neuromuscular junction in vivo. J Cell Sci. 2017;130(10):1752–9. 10.1242/jcs.198358. 28364093 10.1242/jcs.198358 PMC5450190

[6] Fradkin LG, Baines RA, van der Plas MC, Noordermeer JN. The dystrophin Dp186 isoform regulates neurotransmitter release at a central synapse in Drosophila. J Neurosci. 2008;28(19):5105–14. 10.1523/JNEUROSCI.4950-07.2008. 18463264 10.1523/JNEUROSCI.4950-07.2008 PMC2408742

[7] Van der Plas MC, Pilgram GS, Plomp JJ, de Jong A, Fradkin LG, Noordermeer JN. Dystrophin is required for appropriate retrograde control of neurotransmitter release at the Drosophila neuromuscular junction. J Neurosci. 2006;26(1):333–44. 10.1523/JNEUROSCI.4069-05.2006. 16399704 10.1523/JNEUROSCI.4069-05.2006 PMC6674336

[8] Howard PL, Dally GY, Wong MH, Ho A, Weleber RG, Pillers DA, et al. Localization of dystrophin isoform Dp71 to the inner limiting membrane of the retina suggests a unique functional contribution of Dp71 in the retina. Hum Mol Genet. 1998;7(9):1385–91. 10.1093/hmg/7.9.1385. 9700191 10.1093/hmg/7.9.1385

[9] Ueda H, Baba T, Ohno S. Current knowledge of dystrophin and dystrophin-associated proteins in the retina. Histol Histopathol. 2000;15(3):753–60. 10.14670/HH-15.753. 10963120 10.14670/HH-15.753

[10] Pillers DA, Bulman DE, Weleber RG, Sigesmund DA, Musarella MA, Powell BR, et al. Dystrophin expression in the human retina is required for normal function as defined by electroretinography. Nat Genet. 1993;4(1):82–6. 10.1038/ng0593-82. 8513332 10.1038/ng0593-82

[11] Fitzgerald KM, Cibis GW, Giambrone SA, Harris DJ. Retinal signal transmission in Duchenne muscular dystrophy: evidence for dysfunction in the photoreceptor/depolarizing bipolar cell pathway. J Clin Invest. 1994;93(6):2425–30. 10.1172/JCI117250. 8200977 10.1172/JCI117250 PMC294450

[12] Wersinger E, Bordais A, Schwab Y, Sene A, Benard R, Alunni V, et al. Reevaluation of dystrophin localization in the mouse retina. Invest Ophthalmol Vis Sci. 2011;52(11):7901–8. 10.1167/iovs.11-7519. Published 2011 Oct 7. 21896869 10.1167/iovs.11-7519

[13] Bordais A, Bolaños-Jimenez F, Fort P, Varela C, Sahel JA, Picaud S, et al. Molecular cloning and protein expression of Duchenne muscular dystrophy gene products in porcine retina. Neuromuscul Disord. 2005;15(7):476–87. 10.1016/j.nmd.2005.03.011. 15941659 10.1016/j.nmd.2005.03.011

[14] Kameya S, Araki E, Katsuki M, Mizota A, Adachi E, Nakahara K, et al. Dp260 disrupted mice revealed prolonged implicit time of the b-wave in ERG and loss of accumulation of beta-dystroglycan in the outer plexiform layer of the retina. Hum Mol Genet. 1997;6(13):2195–203. 10.1093/hmg/6.13.2195. 9361023 10.1093/hmg/6.13.2195

[15] Barboni MTS, Joachimsthaler A, Roux MJ, Nagy ZZ, Ventura DF, Rendeon A, et al. Retinal dystrophins and the retinopathy of Duchenne muscular dystrophy. Prog Retin Eye Res. 2023;95:101137. 10.1016/j.preteyeres.2022.101137. 36404230 10.1016/j.preteyeres.2022.101137

[16] Ricotti V, Jägle H, Theodorou M, Moore AT, Muntoni F, Thompson DA. Ocular and neurodevelopmental features of Duchenne muscular dystrophy: a signature of dystrophin function in the central nervous system. Eur J Hum Genet. 2016;24(4):562–8. 10.1038/ejhg.2015.135. 26081639 10.1038/ejhg.2015.135 PMC4929863

[17] D’Souza VN, Nguyen TM, Morris GE, Karges W, Pillers DA, Ray PN. A novel dystrophin isoform is required for normal retinal electrophysiology. Hum Mol Genet. 1995;4(5):837–42. 10.1093/hmg/4.5.837. 7633443 10.1093/hmg/4.5.837

[18] Blank M, Koulen P, Blake DJ, Kröger S. Dystrophin and beta-dystroglycan in photoreceptor terminals from normal and mdx3Cv mouse retinae. Eur J Neurosci. 1999;11(6):2121–33. 10.1046/j.1460-9568.1999.00636.x. 10336681 10.1046/j.1460-9568.1999.00636.x

[19] Omori Y, Araki F, Chaya T, Kajimura N, Irie S, Terada K, et al. Presynaptic dystroglycan-pikachurin complex regulates the proper synaptic connection between retinal photoreceptor and bipolar cells. J Neurosci. 2012;32(18):6126–37. 10.1523/JNEUROSCI.0322-12.2012. 22553019 10.1523/JNEUROSCI.0322-12.2012 PMC6622127

[20] Catalani E, Bongiorni S, Taddei AR, Mezeeti M, Silvestri F, Coazzoli M, et al. Defects of full-length dystrophin trigger retinal neuron damage and synapse alterations by disrupting functional autophagy. Cell Mol Life Sci. 2021;78(4):1615–36. 10.1007/s00018-020-03598-5. 32749504 10.1007/s00018-020-03598-5 PMC7904721

[21] Ueda H, Kobayashi T, Mitsui K, Tsurugi K, Tsukahara S, Ohno S. Dystrophin localization at presynapse in rat retina revealed by immunoelectron microscopy. Invest Ophthalmol Vis Sci. 1995;36(11):2318–22. 7558727

[22] Ueda H, Baba T, Terada N, Kato Y, Tsukahara S, Ohno S. Dystrophin in rod spherules; submembranous dense regions facing bipolar cell processes. Histochem Cell Biol. 1997;108(3):243–8. 10.1007/s004180050164. 9342618 10.1007/s004180050164

[23] Ueda H, Kato Y, Baba T, Terada N, Fujii Y, Tsukahara S, et al. Immunocytochemical study of dystrophin localization in cone cells of mouse retinas. Invest Ophthalmol Vis Sci. 1997;38(8):1627–30. 9224291

[24] Frigeri A, Nicchia GP, Repetto S, Bado M, Minetti C, Svelto M. Altered aquaporin-4 expression in human muscular dystrophies: a common feature? FASEB J. 2002;16(9):1120–2. 10.1096/fj.01-0797fje. 12039847 10.1096/fj.01-0797fje

[25] Persiconi I, Cosmi F, Guadagno NA, Lupo G, De Stefano ME. Dystrophin Is Required for the Proper Timing in Retinal Histogenesis: A Thorough Investigation on the mdx Mouse Model of Duchenne Muscular Dystrophy. Front Neurosci. 2020;14:760. 10.3389/fnins.2020.00760. Published 2020 Aug 31. 32982660 10.3389/fnins.2020.00760 PMC7487415

[26] Lombardi L, De Stefano ME, Paggi P. Components of the NGF signaling complex are altered in mdx mouse superior cervical ganglion and its target organs. Neurobiol Dis. 2008;32(3):402–11. 10.1016/j.nbd.2008.07.021. 18725298 10.1016/j.nbd.2008.07.021

[27] Lombardi L, Persiconi I, Gallo A, Hoogenraad CC, De Stefano ME. NGF-dependent axon growth and regeneration are altered in sympathetic neurons of dystrophic mdx mice. Mol Cell Neurosci. 2017;80:1–17. 10.1016/j.mcn.2017.01.006. 28161362 10.1016/j.mcn.2017.01.006

[28] García-Cruz C, Aragón J, Lourdel S, Annan A, Roger J, Montanez C, et al. Tissue- and cell-specific whole-transcriptome meta-analysis from brain and retina reveals differential expression of dystrophin complexes and new dystrophin spliced isoforms. Hum Mol Genet. 2023;32(4):659–76. 10.1093/hmg/ddac236. 36130212 10.1093/hmg/ddac236 PMC9896479

[29] Oshima J, Magner DB, Lee JA, Breman AM, Schmitt ES, White LD, et al. Regional genomic instability predisposes to complex dystrophin gene rearrangements. Hum Genet. 2009;126(3):411–23. 10.1007/s00439-009-0679-9. 19449031 10.1007/s00439-009-0679-9

[30] Bucher F, Friedlander MSH, Aguilar E, Kurihara T, Krohne TU, Usui Y, et al. The long dystrophin gene product Dp427 modulates retinal function and vascular morphology in response to age and retinal ischemia. Neurochem Int. 2019;129:104489. 10.1016/j.neuint.2019.104489. 31199961 10.1016/j.neuint.2019.104489

[31] So K, Shinoda K, Watanabe E, Mashiko T, Mizota A. Vitrectomy for proliferative retinopathy in patient with advanced duchenne muscular dystrophy. Retin Cases Brief Rep. 2012;6(2):142–4. 10.1097/ICB.0b013e3182160913. 25390945 10.1097/ICB.0b013e3182160913

[32] Park SH, Lee YH, Han JW, Rim TH. Proliferative retinopathy developed in a duchenne muscular dystrophy patient with normal cardiac function. J Retina. 2019;4(1):36–9. 10.21511/jr.2019.4.1.36.

[33] Runge AK, Remlinger J, Abegg M, Ferrazzini T, Brügger D, Weight- Usinger K, et al. Retinal layer segmentation in a cohort of healthy children via optical coherence tomography. PLoS ONE. 2022;17(11):e0276958. 10.1371/journal.pone.0276958. Published 2022 Nov 3. 36327296 10.1371/journal.pone.0276958 PMC9632928

[34] Banc A, Ungureanu MI. Normative data for optical coherence tomography in children: a systematic review. Eye (Lond). 2021;35(3):714–38. 10.1038/s41433-020-01177-3. 32929184 10.1038/s41433-020-01177-3 PMC8027201

[35] Kersey JP, Broadway DC. Corticosteroid-induced glaucoma: a review of the literature. Eye (Lond). 2006;20(4):407–16. 10.1038/sj.eye.6701895. 15877093 10.1038/sj.eye.6701895

[36] Bushby K, Finkel R, Birnkrant DJ, Case LE, Clemens PR, Cripe L, et al. Diagnosis and management of Duchenne muscular dystrophy, part 1: diagnosis, and pharmacological and psychosocial management. Lancet Neurol. 2010;9(1):77–93. 10.1016/S1474-4422(09)70271-637. 19945913 10.1016/S1474-4422(09)70271-6

[37] Valamanesh F, Torriglia A, Savoldelli M, Gandolphe C, Jeanny JC, BenEzra D, et al. Glucocorticoids induce retinal toxicity through mechanisms mainly associated with paraptosis. Mol Vis. 2007;13:1746–57. 17960113

[38] Gürağaç FB, Totan Y, Güler E, Tenlik A, Ertuğrul İG. Normative Spectral Domain Optical Coherence Tomography Data in Healthy Turkish Children. Semin Ophthalmol. 2017;32(2):216–22. 10.3109/08820538.2015.1053625. 26795877 10.3109/08820538.2015.1053625

[39] Mohammad Salih PA. Evaluation of peripapillary retinal nerve fiber layer thickness in myopic eyes by spectral-domain optical coherence tomography. J Glaucoma. 2012;21(1):41–4. 10.1097/IJG.0b013e3181fc8053. 21173707 10.1097/IJG.0b013e3181fc8053

